# The House Surgeon and the Stethoscope

**Published:** 1909-01-23

**Authors:** 


### Resident Medical Officers' Department.

### THE HOUSE SURGEON AND THE STETHOSCOPE.

In large general hospitals, proportionately staffed, curious traditions of specialisation are sometimes to be observed among the resident medical and surgical officers. Arbitrary distinctions between cases that are surgical and cases that are medical exist, of course, in every hospital, and have their place among the unwritten laws of each institution. For the smooth working between the various departments some definite means of classification is clearly necessary, even if it occasionally appears fantastic or ridiculous; and in actual practice the lines drawn between medical, surgical, and gynæcological cases are seldom quite so hard and fast as they seem at first sight. It is not so much with traditions of this kind that we propose to concern ourselves in this article, as with other traditions of a less definite character which influence the individual attitude and conduct of certain house officers. Some of these customs are more apparent than real, and some have their foundations buried in logic and necessity. But the one which we have especially in mind has nothing that can be advanced in its favour, and little that can reasonably be said in its defence. We refer to the same attitude of house surgeons towards the stethoscope.

In days gone by, when the surgeon was frequently without any medical degree, and the physician was as often as not unqualified in surgery, certain distinctions between their respective provinces were not only expedient but inevitable. Nowadays, when no man can be registered at all who is not qualified by examination to practise medicine, surgery, and midwifery, there is still much specialisation of function, but, on the whole, the point of view of the profession in this respect is considerably wider and less absurd. The house surgeon, no less than the consulting surgeon, cannot be for ever practising upon the stethoscope, or experimenting with elaborate prescriptions; his time is too fully occupied with other and more pressing affairs. But too often the attention given by the house surgeon to the thorax and its contents seems as perfunctory as his attitude towards it seems contemptuous. This outward scorn of medical methods of examination is naturally more marked in those house surgeons whose exclusive goal is surgery; but it is also to be seen—usually, let us hope, as a temporary phenomenon—in those who are destined for general practice.

That kind of specialisation which keeps a man concentrated upon his own task and bids him not to meddle with other people's business is good; but the whole body is the house surgeon's business, and not merely the parts thereof which lie above the clavicles and below the diaphragm; and he is indeed but a poor sort of surgeon who is not on occasion something more than an amateur physician.

It sounds incredible, but it is none the less true, that highly competent house surgeons, bristling with common sense, are occasionally heard to boast that they never carry a stethoscope, but borrow one from somebody if there is a heart or a lung to be examined. If this implied merely that auscultation in their opinion is the least important part of the physical examination of the chest their point of view would command our respect and sympathy. But the opposite is more commonly what they mean. In the surgical wards a few vigorous flourishes with the stethoscope are often considered all that is necessary for a thorough investigation of the thorax and its contents. That is the least that is demanded of the house surgeon before a patient is sent up to the theatre for anæsthetisation, and that is often the most that is given to the surgical patient during his stay in hospital.

It must be owned that there is seldom time in the busy house surgeon's day for more than the briefest examination of heart and lungs; but it is not the actual fact, so much as the mental attitude which too often underlies it, that we would hold up to rebuke. The house surgeon who, during his term of office, resolutely abstains from any procedure not labelled "strictly surgical" is cultivating a narrow frame of mind almost as dangerous as the habit still found in certain physicians of speaking and acting as though the surgeon were an inferior order of mechanic, whose function (like that of the chiropodist) is to come and operate at the bidding of the physician.

As an antidote to extreme specialisation of the duties of house surgeons and house physicians, the custom in certain great hospitals of allowing the latter to resect ribs for empyemata and to perform tracheotomies, has much to commend it. The origin of this custom depends, of course, on other considerations, principally those of expediency; but none the less it serves to remind both the house surgeon and the house physician that each is a qualified practitioner of medicine, and that, in hospital at least, the distinction between medical and surgical practice exists solely for the promotion of an economic division of labour.

